# The brain correlates of the effects of monetary and verbal rewards on intrinsic motivation

**DOI:** 10.3389/fnins.2014.00303

**Published:** 2014-09-18

**Authors:** Konstanze Albrecht, Johannes Abeler, Bernd Weber, Armin Falk

**Affiliations:** ^1^Department of Education, Cognition, and Communication, Institute of Psychology, RWTH Aachen UniversityAachen, Germany; ^2^Department of Economics, University of OxfordOxford, UK; ^3^Center for Economics and Neuroscience, University of BonnBonn, Germany; ^4^Department of Epileptology, University Hospital of BonnBonn, Germany; ^5^Department of Economics, University of BonnBonn, Germany

**Keywords:** intrinsic motivation, crowding-out, monetary rewards, verbal rewards, fMRI

## Abstract

Apart from everyday duties, such as doing the laundry or cleaning the house, there are tasks we do for pleasure and enjoyment. We do such tasks, like solving crossword puzzles or reading novels, without any external pressure or force; instead, we are intrinsically motivated: we do the tasks because we enjoy doing them. Previous studies suggest that external rewards, i.e., rewards from the outside, affect the intrinsic motivation to engage in a task: while performance-based monetary rewards are perceived as controlling and induce a business-contract framing, verbal rewards praising one's competence can enhance the perceived self-determination. Accordingly, the former have been shown to *decrease* intrinsic motivation, whereas the latter have been shown to *increase* intrinsic motivation. The present study investigated the neural processes underlying the effects of monetary and verbal rewards on intrinsic motivation in a group of 64 subjects applying functional magnetic resonance imaging (fMRI). We found that, when participants received positive performance feedback, activation in the anterior striatum and midbrain was affected by the nature of the reward; compared to a non-rewarded control group, activation was higher while monetary rewards were administered. However, we did not find a decrease in activation after reward withdrawal. In contrast, we found an increase in activation for verbal rewards: after verbal rewards had been withdrawn, participants showed a higher activation in the aforementioned brain areas when they received success compared to failure feedback. We further found that, while participants worked on the task, activation in the lateral prefrontal cortex was enhanced after the verbal rewards were administered and withdrawn.

## Introduction

In many fields, such as education and the workplace, tangible incentives are used to motivate individuals to learn or to work. However, a large body of psychological and economic research indicates that such incentives do not necessarily enhance, but can also undermine intrinsic motivation (Deci, 1971; Lepper et al., 1973; Ryan et al., 1983; Camerer and Hogarth, 1999; Deci et al., 1999; Irlenbusch and Sliwka, 2005; Mellström and Johannesson, 2008). Empirical studies showed that individuals who received a tangible performance-based reward—such as money—exerted lower effort in a task when the reward was not available anymore (e.g., Deci, 1971; Lepper et al., 1973; Irlenbusch and Sliwka, 2005). In comparison, in a control group that never received a performance-based reward, effort did not decline. The studies further showed that verbal reinforcement can have the opposite effect on participants' engagement in a task: when participants received supportive verbal feedback praising their competence, engagement in the task increased after reward withdrawal.

In their Cognitive Evaluation Theory (CET), Deci and Ryan (1985) explain these findings as follows: CET asserts that two psychological needs underlie intrinsic motivation; the need for self-determination and the need for competence. Accordingly, the effect of a reward on intrinsic motivation depends on how the reward affects these needs, i.e., how it affects the perception of self-determination and the perception of competence. If a reward has a positive effect on these needs by enhancing perceived self-determination and perceived competence, it increases (crowds-in) intrinsic motivation and accordingly the engagement in a task. In contrast, if the effect is negative, perceived self-determination and perceived competence are reduced and intrinsic motivation will be decreased (crowded-out). Whether a reward has a positive or negative effect on these needs depends on its nature: if the reward is perceived as controlling one's behavior (such as monetary rewards), it reduces perceived self-determination, leads to a more external perceived locus of causality and decreases intrinsic motivation. If, on the other hand, the reward is informational and perceived as indicating one's competence (such as verbal reinforcement), it increases intrinsic motivation. Performance-contingent monetary rewards, i.e., rewards that are expected and only awarded when a task is performed successfully, are very likely to be perceived as controlling, since to receive them successful completion of the task is necessary (Deci et al., 1999). In contrast, informational rewards, such as verbal feedback emphasizing an individual's competence in a task, are likely enhancing one's perceived competence and thus lead to an increased intrinsic motivation in the task (Deci et al., 1999).

In neuroscientific research, animal studies were used to investigate and model motivation processes (e.g., Dayan and Balleine, 2002; Shiflett and Balleine, 2010; Wassum et al., 2011). To our knowledge, up to now only one study looked into the effect of tangible rewards on motivation in humans: Murayama et al. (2010) investigated the neural basis of the first part of Deci's and Ryan's (1985) CET, i.e., the crowding-out of intrinsic motivation with monetary rewards. They used a stop-watch task which participants performed over two periods. At first, participants in the monetary reward group received a performance based payment which was withdrawn in the subsequent period. A control group did the same task without any performance-based payment over both periods. They reported an interaction of activation in the anterior part of the striatum and midbrain when participants received success vs. failure feedback: whereas activation was *higher* in these regions for the reward group compared to the control group while rewards were administered, it was *lower* in the reward group after rewards were withdrawn.

Since these regions were associated with a sense of self-agency (Tricomi et al., 2004, 2006; Delgado, 2007; Tricomi and Fiez, 2008; Shohamy, 2011) and responsiveness toward cognitive feedback and rewards (e.g., Delgado et al., 2000; Shohamy et al., 2004; Aron et al., 2004; O'Doherty et al., 2006; D'Ardenne et al., 2008; Haber and Knutson, 2009), respectively, the authors interpreted this as reduced intrinsic motivation of the reward group to engage in the task after reward withdrawal. They further observed that activation in the right lateral prefrontal cortex (rLPFC) showed the same interaction effect during the cue phase, i.e., when participants learned whether an interesting or boring task was coming next. The rLPFC is assumed to be involved in the cognitive processes underlying the motivation to achieve goals (Duncan et al., 1996; Leon and Shadlen, 1999; Miller and Cohen, 2001; Wager and Smith, 2003; Bunge, 2004), and that this motivation is modulated by reward sensitivity (Jimura et al., 2010). It could hence signal the motivation to exert effort in an upcoming task.

In the present functional magnetic resonance study, we, similarly to Murayama et al. (2010), investigated the administration and withdrawal of monetary rewards on brain activation. We additionally investigated the effect of verbal rewards in a comparable way.

Like Murayama et al. (2010), we expected the anterior striatum and midbrain to be responsive to reward feedback. Several studies suggest that the anterior striatum plays a role in the perception of agency (Tricomi et al., 2004, 2006; Delgado, 2007; Tricomi and Fiez, 2008) and is responsive to both monetary and social rewards (Izuma et al., 2008). For example, Delgado (2007) reported that activation to feedback in the striatum was higher when rewards were contingent on behavior, suggesting an association with self-agency. Further, the striatum was found to be more highly activated when success was not determined randomly (i.e., by luck) but by informed choice (Tricomi and Fiez, 2008). CET claims that monetary and verbal rewards affect the perception of self-determination (Deci and Ryan, 1985), which accordingly may be reflected in anterior striatum activation. Midbrain structures have been shown to be connected to the striatum (Haber et al., 2000; Kahnt et al., 2009), to be responsive to monetary and non-monetary rewards and are supposed to be involved in the subjective valuation of rewards (e.g., Shohamy et al., 2004; Aron et al., 2004; O'Doherty et al., 2006; D'Ardenne et al., 2008). For example, an increased activation in the midbrain was not only found in the presence of monetary rewards (Shohamy et al., 2004) but also when cognitive feedback, informing a person that her response was correct, was given (Aron et al., 2004). Further, O'Doherty et al. (2006) showed that midbrain activation corresponds to reward preferences; the higher a participant ranked a certain juice stimulus, the higher was the midbrain's activation in response to delivery of this stimulus. Thus, midbrain activation might reflect high valuation of success feedback.

Specifically, compared to the control group without rewards, we expected activation in the anterior striatum and midbrain to be higher while monetary rewards were administered and lower after rewards were withdrawn. This would be in line with both performance measures of behavioral studies and the imaging results by Murayama et al. (2010), suggesting a neural crowding-out effect of monetary rewards.

In the group that received verbal reinforcement, we expected activation to be higher after reinforcement was withdrawn compared to the control group, which would suggest a crowding-in effect of verbal rewards. We did not have a hypothesis about differences in activation in the second period, i.e., while verbal reinforcement was administered, since neither CET (Deci and Ryan, 1985) predicts any differences here, nor were differences in actual performance in this period between the verbal reinforcement group and the control group reported (Deci, 1971).

While participants executed the task (i.e., before they received task feedback), we expected the rLPFC to be engaged (Duncan et al., 1996; Miller and Cohen, 2001). We hypothesized that activation in this area would reflect the exerted effort, leading to a higher activation while monetary rewards were administered, but to a lower activation after reward withdrawal. In contrast, we expected effort and hence activation to be higher after verbal rewards were withdrawn. We expected neural activation to be reflected behaviorally in the number of correctly solved items during a period, providing a behavioral measure of intrinsic motivation (Deci, 1971; Lepper et al., 1973).

## Materials and methods

### Participants

Sixty four participants (38 female, range 18–34 years, mean 24.16 years, SD 3.26 years) without any history of neurological or psychiatric disease participated in the study. Seven additional participants had to be excluded because of excessive head movement. All subjects were right-handed according to the Edinburgh Handedness Scale.

### Ethics statement

All participants gave written informed consent before the study. The experimental procedure of the study was approved by the local ethics committee of the University hospital of Bonn. Data were handled anonymously.

### Materials

**Figure 1:**
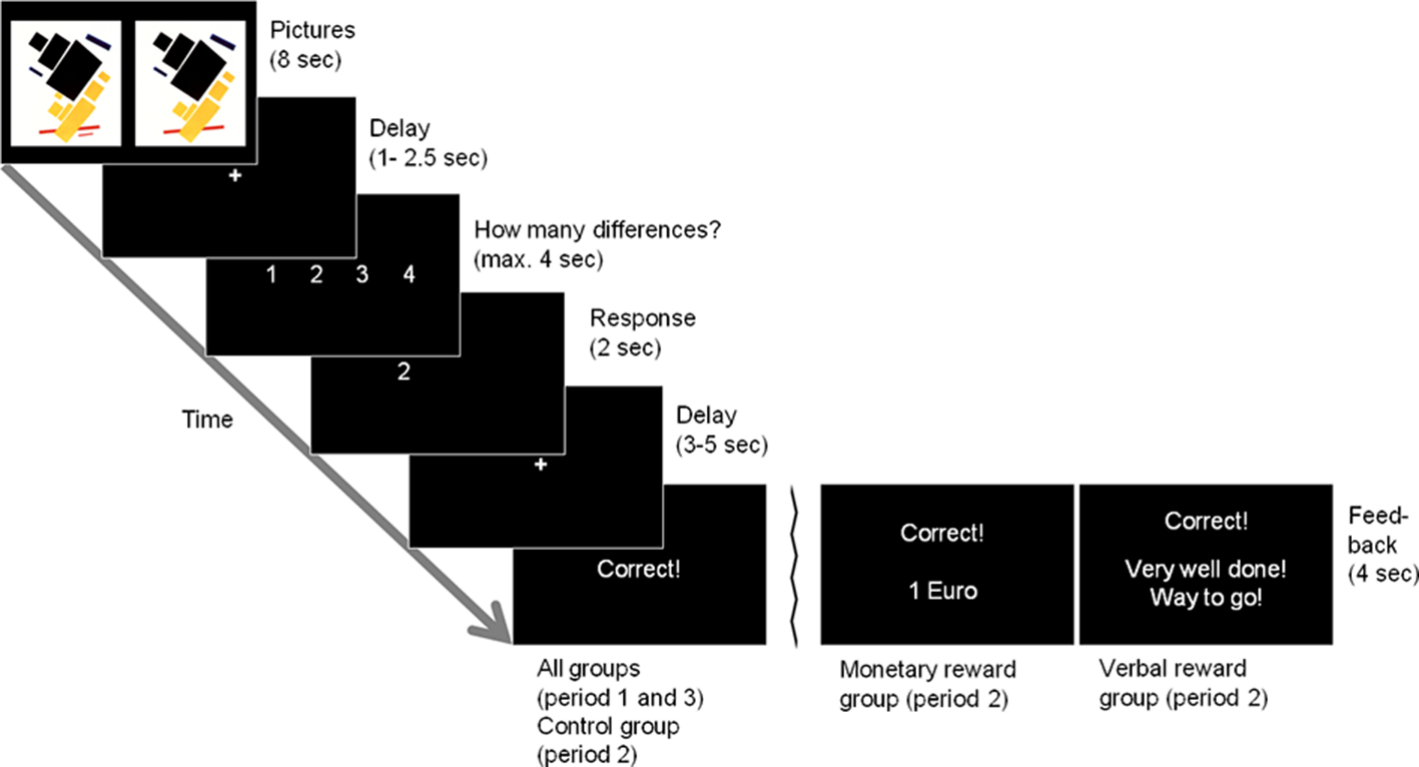
**Example trial**. Two picture stimuli are presented simultaneously. The subjects have to find the number of differences between the stimuli. There are two differences between the original picture stimulus (top panel, left) and the manipulated version (top panel, right) in this example, in which a red and a black bar are missing. Feedback for a correct solution is displayed as given in the control group and in period 1 and 3 in the reward groups (leftmost bottom panel). The two alternative feedback panels display the feedback given in the monetary and verbal reward groups in period 2 (middle and rightmost bottom panels, respectively).

In the before-mentioned behavioral studies, mostly interesting tasks have been used, such as doing puzzles or drawings (Deci, 1971; Lepper et al., 1973). We used picture puzzles in our study; in each trial, participants were presented with a relatively unknown modern art picture that was displayed twice. One was the original version, the other one showed 1–4 small deviations from the original version (see Figure 1 for an example). Participants had to find these differences and indicate their number. We consider this an interesting task since many people do this for fun in everyday life and hence should be intrinsically motivated to do such puzzles. In total, 120 such pictures (40 in each of three periods) were presented to each participant.

### Procedure

Upon arriving, each participant received written instructions of the first period of the experiment. Subjects were randomly assigned to one of three groups: a monetary reward group (22 subjects, 15 female), a verbal reward group (23 subjects, 11 female) and a control group (19 subjects, 12 female). Subjects were unaware of the existence of different groups. Then subjects entered the scanner and, after five training trials, performed the first period with 40 picture puzzles (being the same for all groups). Participants knew that there would be two subsequent periods, but had no further information about their content.

#### Period 1 (baseline)

In the fMRI scanner, each participant faced the first 40 picture puzzles in a random order. In each trial the pictures were displayed for 8 s, before they were replaced by a fixation cross (1–2.5 s) until a response could be entered. Afterwards, a short response feedback was given (2 s), and after a brief delay (3–5 s), feedback about the correctness of the response was given (4 s). Figure 1 displays an overview of the course of one trial. All participants did this first period as a baseline condition, i.e., it was the same for all participants and could later be used to determine differences in the individual baseline intrinsic motivation concerning the task. Afterwards, participants were asked via intercom whether they needed a break. All participants wanted to go on with the second period right away.

#### Period2 (monetary/verbal/control manipulation)

For the control group, the course of a trial did not differ to that of period 1. The monetary reward group additionally received the information that now they would get paid € 1 for each puzzle they solved correctly. If the response was correct in the verbal reward group, “Very well done! Way to go!” was displayed during feedback presentation.

After the second period ended, participants left the fMRI scanner and took a break of 10 to 15 min before going back in. This was made to minimize possible effects (e.g., exhaustion) that different effort levels (which might have arisen in period 2 due to treatment differences) might otherwise have had on performance in period 3.

#### Period 3

It was stated clearly to the participants of the monetary reward group that no monetary rewards would be provided anymore. It might have been more natural to not announce this reward withdrawal and thus not to direct subjects' attention to it. Yet, not announcing it might have led to false expectations, i.e., to the expectation that there still might be a performance-based reward in the end which was just not announced in each single trial anymore. In the verbal reward group, this was no concern, since verbal rewards did not lead to any tangible consequences, but rather were to be “consumed” the moment they appeared. Thus, the verbal reward group simply did not receive any additional verbal feedback anymore, without further announcement.

Each period lasted about 17 min. Pictures were presented in random order and counterbalanced between subjects for periods 1 and 3. Pictures in period 2 were presented in random order but stayed the same across subjects. After period 3, all subjects indicated how much fun they had solving the picture puzzles on a 7-point Likert scale. See Table 1 for an overview of the procedure.

**Table 1:** **Feedback given in the different periods to the different groups of participants**.

	**Monetary reward group**	**Verbal reward group**	**Control group**
Period 1	Correct vs. incorrect	Correct vs. incorrect	Correct vs. incorrect
Period 2	Correct “**1 Euro**” vs. Incorrect “**0 Euro**”	Correct “**Very well done, way to go!**” vs. Incorrect	Correct vs. incorrect
Period 3	Correct vs. incorrect	Correct vs. incorrect	Correct vs. incorrect

### fMRI data acquisition

Scanning was performed on a 1.5-Tesla (T) Avanto Scanner(Siemens), by using a standard 8-channel head coil. Slices were in axial orientation and covered all of the brain including the midbrain but not the entire cerebellum. Scan parameters were as follows: slice thickness, 2 mm; interslice gap, 1 mm; echo time (TE), 45 ms; repetition time (TR), 2.83 s. The scanning was performed in 3 sessions with 40 trials each for ~17 min each, resulting in an overall scanning time of ~ 51 min and ~1, 180 scans. (Data is available upon request. Please contact the corresponding author at albrecht@psych.rwth-aachen.de).

### fMRI data analysis

fMRI data analysis was performed by using Statistical Parametric Mapping 5 (SPM5, www.fil.ion.ucl.ac.uk/spm/). For preprocessing, functional images were realigned to the first image of the first session of each time series and again realigned to the mean image after first realignment. Images were then normalized to the canonical EPI template used in SPM5, and smoothed with an 8-mm Gaussian kernel. After normalization images were resampled to a voxel size of 3 × 3 × 3 mm.

For modeling the blood oxygen-level dependent (BOLD) response, five regressors were defined for each period: presentation of correctly solved pictures (PC), presentation of incorrectly solved pictures (PI), response (R) presentation of success feedback (FBS), and presentation of failure feedback (FBF). The onsets for our regressors can be inferred from Figure 1 like this: the onset of PC/PI was when the picture puzzle was presented (top panel). This presentation lasted for 8 s, until the display of the picture puzzles was replaced by a fixation cross. R was onset when the fixation cross (1–2.5 s) was replaced by the display of the possible responses and ended after the given response (indicated by a button press) was displayed for 2 s (panels 3 + 4 from top). After the display of another fixation cross (3–5 s), the feedback (FBS/FBF) was onset and displayed for 4 s (bottom panels). Parameter images for the contrasts for each single condition were generated for each subject and were then subjected to a second-level random effects analysis.

We conducted second-level whole-brain ANOVA with within-subject factor period and between-subject factor group. We tested interaction effects of feedback [always using contrasts of success minus failure feedback (FBS-FBF)] corrected for individual baseline differences for period and group separately for the two reward groups., i.e., we conducted an ANOVA for period (2–1 vs. 3–1) and group (monetary reward vs. control) as well as for period (2–1 vs. 3–1) and group (verbal reward vs. control). We further tested whether results would change when not controlling for baseline activation (i.e., when not subtracting activation in period 1). Additionally, we ran a period (3–2) × group (monetary reward vs. control) ANOVA.

Analogously, we conducted the same ANOVAs for picture presentation, i.e., while participants saw the picture puzzles and tried to find the correct number of differences between the two pictures. Only trials of correctly solved picture puzzles (regressor PC) were included.

Reported *p*-values are two-sided, if not stated otherwise. All reported clusters for uncorrected comparisons consist of a minimum of 10 voxels.

We conducted small volume corrections and extracted parameter estimates for our regions of interest from a 12 mm sphere centered on the peak voxel of the anterior striatum (21 20 −2, −21 20 1) and midbrain (−9 −7 −11) activation reported by Murayama et al. (2010), using Marsbar (www.marsbar.sourceforge.net/). We further conducted the same analyses for the rLPFC [39 41 40 (Murayama et al., 2010)] for picture presentation.

## Results

### Behavioral results

**Figure 2:**
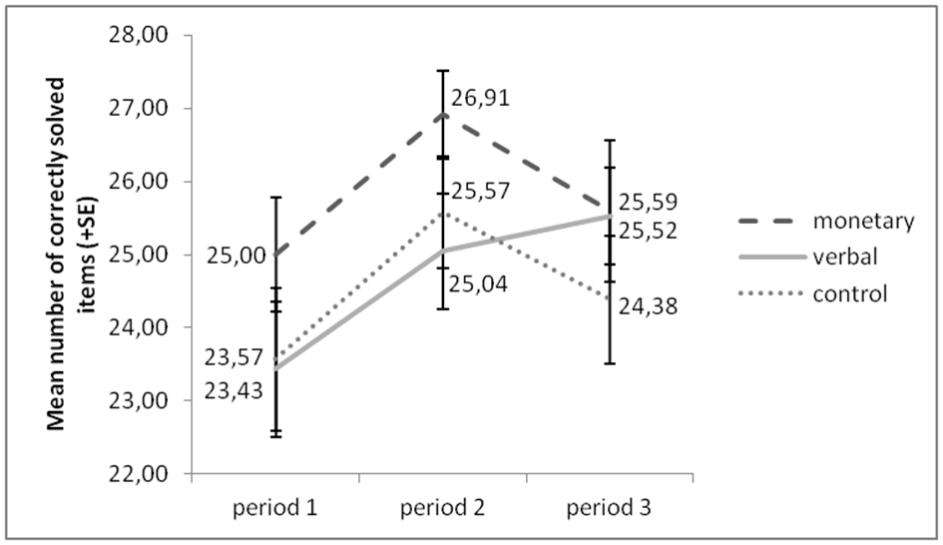
**Performance increased from period 1 to period 2 in all groups, but from period 1 to 3 in the verbal reward group only**.

Figure 2 presents an overview of participants' performance (correctly solved items) over all periods. An ANOVA with the factors group (monetary, verbal, control) and period (1–3) yielded a significant main effect of period [*F*_(2, 122)_ = 6.732, *p* = 0.002]. The effect of group [*F*_(2, 61)_ = 1.751, *p* = 0.184] and the interaction effect of the two factors [*F*_(4, 122)_ = 1.004, *p* = 0.408] were not significant. *Post-hoc* analyses (Sidak-corrected for multiple comparisons) revealed a significant increase of performance from period 1 to 2, irrespective of group (*p* = 0.003). This suggests general training effects irrespective of our treatment manipulation. Alternatively, since only the presentation of pictures in periods 1 and 3 was counterbalanced between subjects, it is possible that the picture puzzles in period 2 were slightly easier to solve than those in the other two periods.

Only in the verbal reward group did performance increase further in period 3 (*p* = 0.040), whereas in the other two groups performance did not increase significantly from period 1 to 3 (monetary: *p* = 0.862; control: *p* = 0.968). However, a univariate ANOVA with group as independent variable and performance increase as dependent variable yielded that this increase was not significantly stronger in the verbal reward group than in the other two groups (verbal vs. monetary: *p* = 0.500; verbal vs. control: *p* = 0.414).

After the experiment, subjects indicated how much fun they had solving the picture puzzles on a 7-point Likert scale. On average, subjects in all three groups stated that it was fun (total: 4.92, monetary: 5.36, verbal: 4.52, control: 4.89). A univariate ANOVA yielded no significant differences in fun ratings between groups [*F*_(2, 61)_ = 1.784, *p* = 0.177].

A systematic summary of statistics for all *post-hoc* main and interaction effects can be found in Tables A1a,b in the supplementary material.

### Imaging results

#### Baseline period 1

We ran an ANOVA contrasting success and failure feedback in period 1. Since in period 1, all groups received the same feedback (success vs. failure) without any monetary or verbal rewards, we analyzed the data across groups.

In line with previous literature (Daniel and Pollmann, 2010), we find higher activation in success compared to failure feedback in the ventral striatum and the medial orbitofrontal cortex (p_FWE_ < 0.05; for coordinates, statistical values and an overview of all areas with activation differences above threshold, see Table A2 in the supplementary material).

In our regions of interest, we find a significant activation difference after small volume correction in the midbrain (p_FWE_ < 0.05). The activation difference does not reach significance in the left anterior striatum (p_FWE_ = 0.063).

#### Task feedback: the monetary reward group

**Figure 3:**
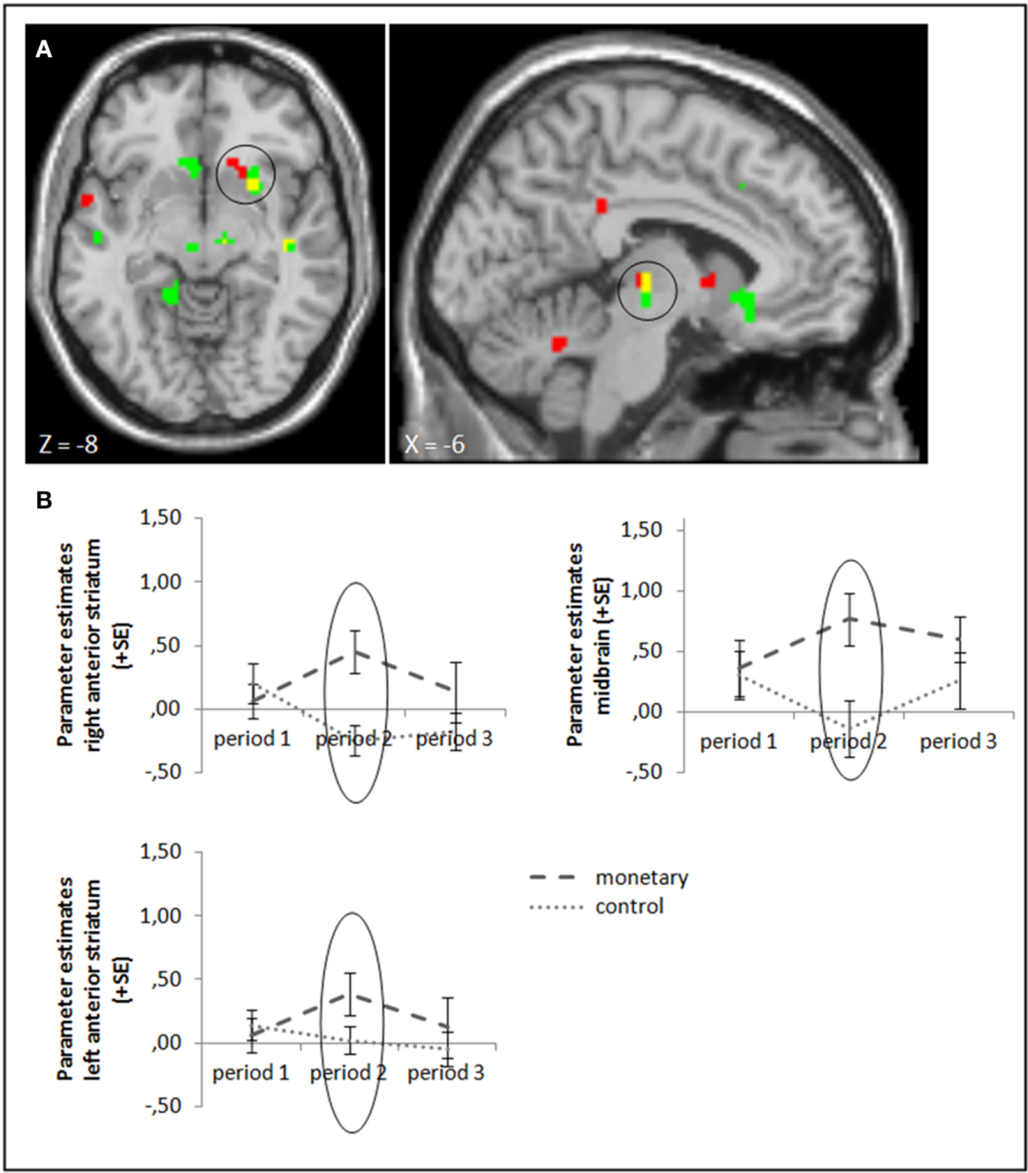
**(A)** Activation for success—failure feedback was significantly higher in the right anterior striatum (left panel) and midbrain (right panel) in the monetary reward group compared to the control group in period 2 (whole brain uncorrected, *p* < 0.001, only clusters with a minimum of 10 activated voxels are shown; small volume corrected, p_FWE_ < 0.05). Activation clusters without baseline correction are shown in green, clusters with baseline correction are shown in red, activation overlaps are shown in yellow. **(B)** Parameter estimates are displayed for illustration.

To test our hypotheses, we ran ANOVA contrasting success and failure feedback between the different groups. We start with comparing the monetary reward group with the control group in periods 2 and 3: in line with Murayama et al. (2010), we found a higher activation in the anterior striatum and midbrain (whole brain uncorrected, *p* < 0.001; small volume corrected, p_FWE_ < 0.05) while monetary rewards were administered. Activation differences in these areas stayed significant when baseline activation of period 1 was subtracted (Figure 3, cf. supplementary material, Table A3). In contrast to Murayama et al. (2010), we did not find any brain area to be activated more strongly in the control compared to the monetary reward group after rewards were withdrawn in period 3, even at a lower threshold (uncorrected, *p* < 0.005). A group × period ANOVA, testing the interaction effect of group and activation differences between periods 3 and 2, also yielded no significant activation differences (uncorrected, *p* < 0.001; small volume corrected, p_FWE_ < 0.05). Tables A3, A5 in the supplementary material give an overview of results for our regions of interest and for all regions, respectively.

#### Task feedback: the verbal reward group

**Figure 4:**
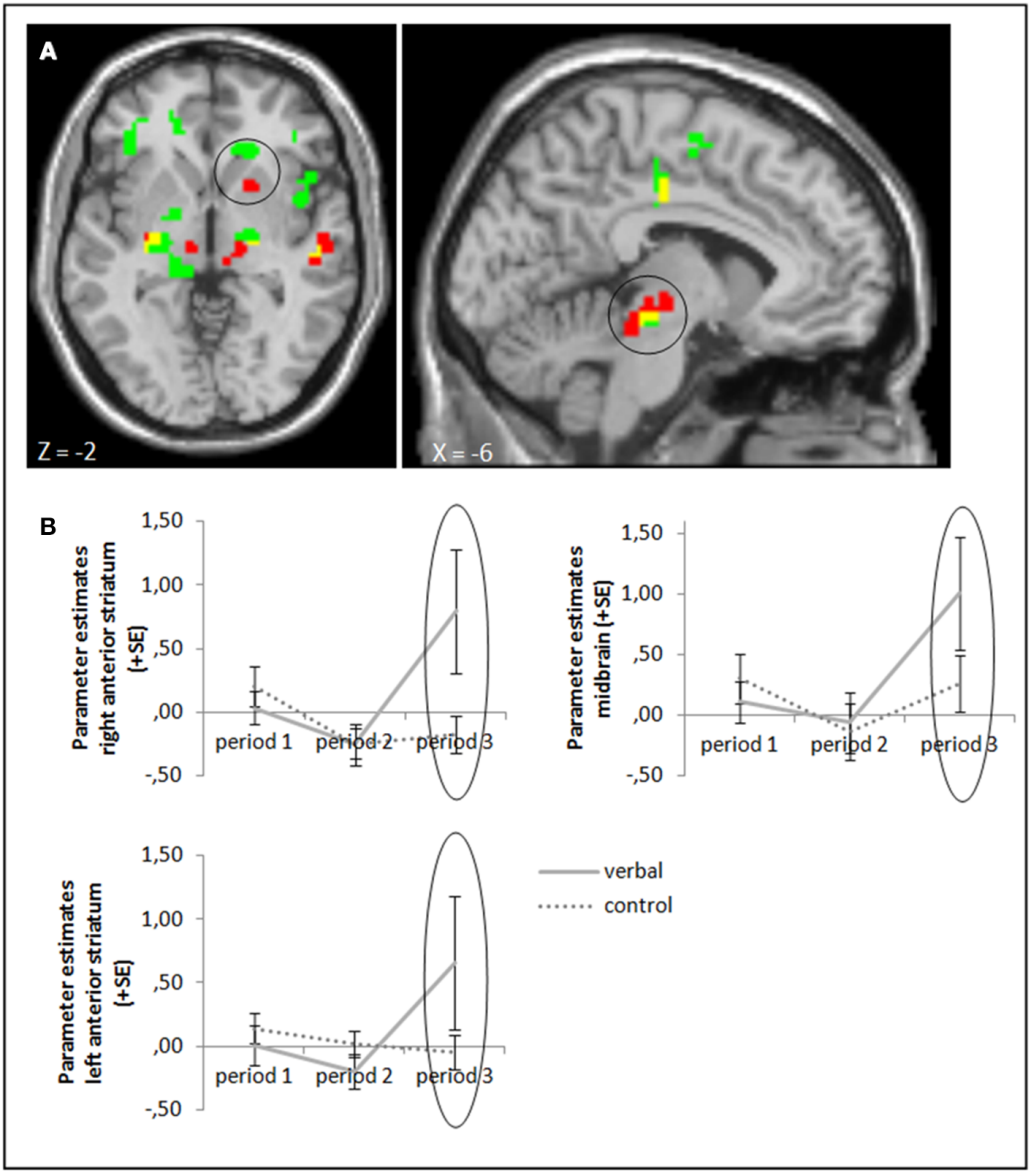
**(A)** Activation for success—failure feedback was significantly higher in the right anterior striatum (left panel) and midbrain (right panel) in the verbal reward group compared to the control group in period 3 (whole brain uncorrected, *p* < 0.001, only clusters with a minimum of 10 activated voxels are shown; small volume corrected, p_FWE_ < 0.05). Activation clusters without baseline correction are shown in green, clusters with baseline correction are shown in red, activation overlaps are shown in yellow. **(B)** Parameter estimates are displayed for illustration.

The ANOVA comparing success—failure feedback in the verbal reward and control groups in period 3 yielded higher activation in our target areas anterior striatum and midbrain (whole brain uncorrected, *p* < 0.001; small volume corrected, p_FWE_ < 0.05) in the group with verbal rewards after reward withdrawal. Activation differences in these areas were also significant when baseline activation of period 1 was subtracted (Figure 4, cf. supplementary material, Table A3). Tables A3, A5 in the supplementary material give an overview of results for our regions of interest and for all regions, respectively.

Self-reported ratings of intrinsic motivation (fun) after period 3 correlated significantly with neural activation (success minus failure feedback) in period 3 only in the verbal reward group (rho = 0.496, *p* = 0.016). The correlation did not reach significance when baseline activation from period 1 was subtracted (rho = 0.294, *p* = 0.173). See Table A7 in the supplementary material for statistical values for the other brain areas and in the other two groups.

#### Picture presentation: the monetary reward group

**Figure 5:**
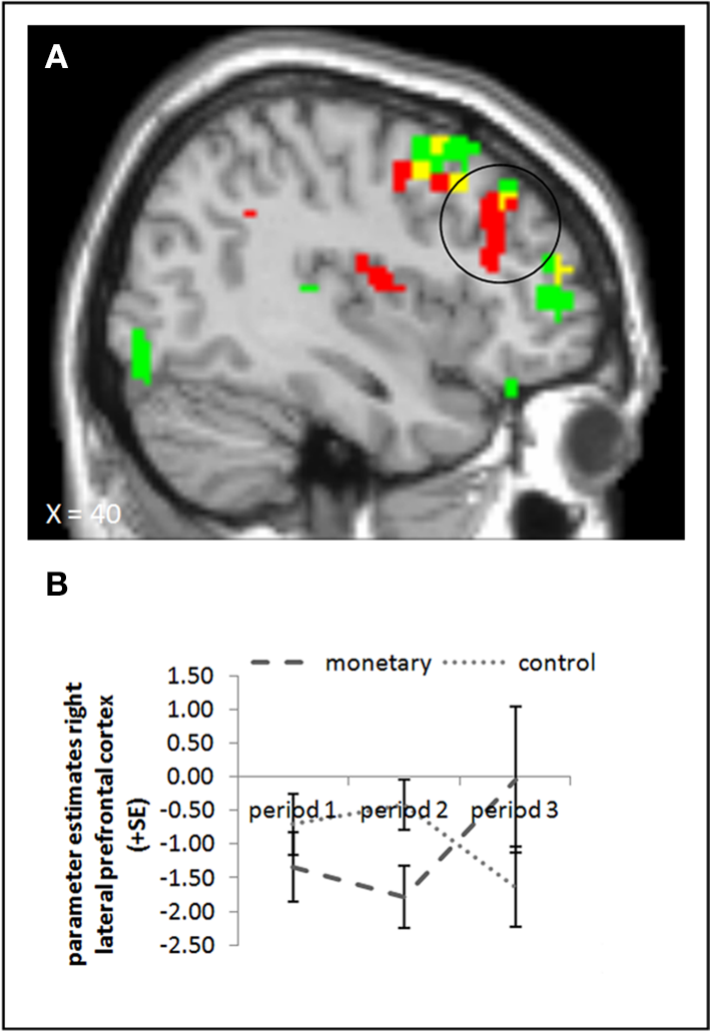
**(A)** Activation for picture presentation was significantly higher in the right lateral prefrontal cortex in the monetary reward group compared to the control group in period 3 (whole brain uncorrected, *p* < 0.001, only clusters with a minimum of 10 activated voxels are shown; small volume corrected, p_FWE_ < 0.05). Activation clusters without baseline correction are shown in green, clusters with baseline correction are shown in red, activation overlaps are shown in yellow. **(B)** Parameter estimates are displayed for illustration.

We conducted the same ANOVA as above for picture presentation, comparing activation in the monetary reward and control group. None of the ANOVA yielded a significant effect in the rLPFC or elsewhere (whole brain uncorrected, *p* < 0.001; small volume corrected; p_FWE_ < 0.05). The interaction contrast of period 3 and period 2 for the control and monetary reward group also showed no significant activation (whole brain uncorrected, *p* < 0.001; small volume corrected; p_FWE_ < 0.05). The parameter estimates suggest that, if there was an effect in this area at all, it runs against our hypotheses: whereas activation in period 2 was *lower* in the monetary reward group than in the control group, in period 3 it seemed to be *higher* in the monetary reward group (Figure 5B). Indeed, activation in period 3 differed significantly between these two groups, also if baseline activation was subtracted (small volume corrected contrast, p_FWE_ < 0.05; Figure 5A, cf. supplementary material, Table A4). However, in period 2 the effect was only significant when baseline activation was *not* taken into account (whole brain uncorrected, *p* < 0.001; small volume corrected, p_FWE_ < 0.05). Further, the interaction of period 3 with period 2 yielded a significant effect (small volume corrected; p_FWE_ < 0.05). Tables A4, A6 in the supplementary material give an overview of results for our regions of interest and for all regions, respectively.

#### Picture presentation: the verbal reward group

**Figure 6:**
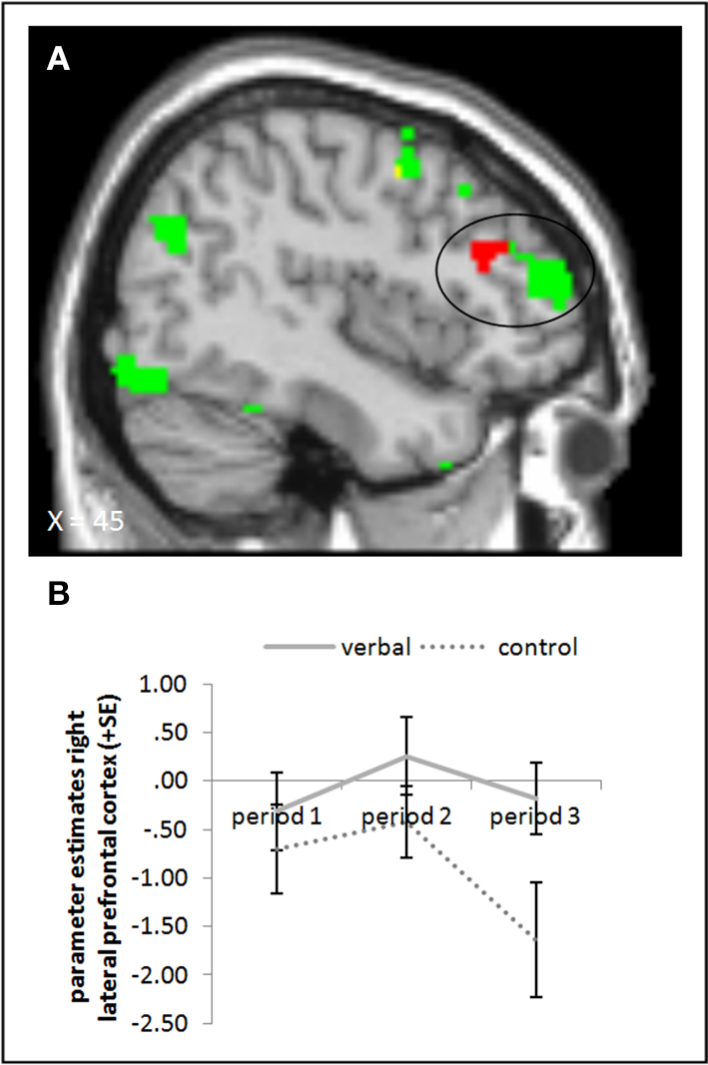
**(A)** Activation for picture presentation was significantly higher in the right lateral prefrontal cortex in the verbal reward group compared to the control group in period 3 (whole brain uncorrected, *p* < 0.001, only clusters with a minimum of 10 activated voxels are shown). Activation clusters without baseline correction are shown in green, clusters with baseline correction are shown in red, activation overlaps are shown in yellow. **(B)** Parameter estimates are displayed for illustration.

The ANOVA comparing the verbal reward and control groups in period 3 during picture presentation yielded higher activation in the rLPFC (whole brain uncorrected, *p* < 0.001) in the group with verbal rewards after reward withdrawal. When we subtracted baseline activation of period 1, we found the activation difference in the rLPFC to be slightly more posterior (Figure 6, cf. supplementary material, Table A4). Further, a small volume correction on a 12 mm sphere centered on the peak voxel from the study of Murayama et al. (2010) did not yield a significant activation difference. Tables A4, A6 in the supplementary material give an overview of results for our regions of interest and for all regions, respectively.

## Discussion

In the present study, we wanted to investigate the neural correlates of crowding-out and crowding-in of intrinsic motivation. To this end, our subjects participated in a series of picture puzzles for three periods receiving feedback about successes and failures in solving the puzzles. In the second period, additionally to success and failure feedback, either monetary rewards (monetary reward group), verbal reinforcement (verbal reward group) or no rewards/reinforcement (control group) were administered. We found that the administration and withdrawal of monetary and verbal rewards influenced brain activation in our regions of interest.

In order to control for baseline brain activation, we subtracted the activation of period 1, in which all three groups solved picture puzzles and received feedback concerning their success in the task. Since we wanted to compare our results to the results of Murayama et al. (2010), who did not run such a baseline treatment, we additionally analyzed our data without controlling for baseline activation. Except for activation in the rLPFC, we could mostly replicate our own results by finding similar activation differences between groups irrespective of baseline activation. A possible explanation for the baseline differences in rLPFC activation could be that cognitive engagement in a task strongly differs between individuals in general, which could for example be due to initial individual differences in intrinsic motivation. A baseline period to control for possible initial variance thus is helpful to rule out such effects.

### No crowding-out effect of monetary rewards

With the present study, we could replicate findings from Murayama et al. (2010) by showing that activation in the anterior striatum and midbrain was higher for success feedback when monetary rewards were involved. This supports the assumption that these areas are indeed involved in subjective valuation and is in line with findings by previous studies that link motivation to monetary rewards and show that these brain areas are more highly activated when additionally to success feedback monetary rewards are administered (Engelmann and Pessoa, 2007; Engelmann et al., 2009; Daniel and Pollmann, 2010; Pessoa and Engelmann, 2010). However, we could not replicate the finding that activation in these areas (or any other brain areas) decreased after monetary rewards were withdrawn. Hence, our data cannot support the crowding-out effect due to monetary rewards. Further, we even found lower activation for the control compared to the monetary reward group in the rLPFC while monetary rewards were administered in period 2, and we found higher activation for the monetary reward compared to the control group in the rLFPC during task presentation in period 3 (i.e., after rewards were withdrawn). We expected this activation to reflect cognitive engagement in the task and hence hypothesized activation to be high when monetary rewards were administered and, due to crowding-out, lower after reward withdrawal. Yet our results indicate that, if at all, motivation was decreased while rewards were administered and increased after their withdrawal. It is difficult to explain the decrease during reward administration. However, the activation we found here was only present when we did not subtract baseline activation, which questions the validity of this result. Although, the parameter estimates extracted from the rLPFC were mostly below the baseline, suggesting a rather weak activation during picture presentation in general. A possible explanation for the finding that activation in the rLPFC was higher in the monetary reward group after reward withdrawal is that participants in the reward group were happy about and grateful for the additional money they just earned and reacted to this by reciprocating and strongly engaging in the task even after performance-based rewards were not paid anymore. We can only speculate about long-term effects, though; it might decline after some time, even leading to a lower activation than observed in the control group. Hence, the long-term effects of withdrawn monetary rewards are still to be investigated in future research.

Although we find activation differences in the same brain areas as Murayama et al. (2010), who can support their results with differences in behavioral engagement, it is not clear whether monetary rewards indeed affected intrinsic motivation in our paradigm. We did not find behavioral differences and baseline brain activation in period 1 is not very strong. On the other hand, subjects' post-experimental fun ratings indicate that subjects indeed had fun solving the picture puzzles, as the mean rating was 4.92 (and even 5.36 for the monetary group alone) on a Likert scale ranging from 1 to 7. Because of these contradicting findings, it remains unclear whether in our task intrinsic motivation was high enough to be negatively affected by monetary rewards.

### The crowding-in effect of verbal rewards

We found an effect of verbal rewards on brain activation: activation in the anterior striatum and midbrain was higher after the administration of verbal rewards than in the control group. Both striatum and midbrain were reported to be involved in the subjective valuation of situations (Schultz et al., 1997; Bayer and Glimcher, 2005; Seymour and McClure, 2008); accordingly these activation differences suggest that individuals have a higher subjective value for succeeding in a task after verbal reinforcement. The activation in the striatum, which was further reported to be involved in evaluating one's influence on an outcome of a situation (Tricomi et al., 2004, 2006), might reflect a higher perceived self-determination—a key component of the crowding-in effect (Deci and Ryan, 1985). Accordingly, verbal reinforcement might increase subjective valuation and perceived self-determination of a task. This last inference is supported also by a significant correlation between striatal activation in period 3 and self-reported fun.

Further, only in this group did performance increase significantly over time, which supports that at least task engagement grew. However, this increase was not significantly larger than that in the control and monetary reward groups. Thus, the inferences from neural activation we made in the above paragraph are only weakly supported by behavioral data and have to be considered with care. Also, although activation in the left striatum in period 3 correlated significantly with self-reported fun ratings, this effect was not robust to subtracting baseline activation from period 1. Taken together with the in general small baseline activation in period 1 and the non-differing post-experimental fun ratings, it remains unclear whether task engagement and accordingly intrinsic motivation were actually influenced by verbal rewards.

The neural evidence for higher task engagement of the verbal reward group during picture presentation is unclear, too; activation in the right rLPFC was higher in the verbal reward compared to the control group in period 3, but the exact location of this activation differences was not identical with the region identified by Murayama et al. (2010). The task used by Murayama and colleagues was different from ours (participants had to press a button to stop a stop watch after 5.00 s as exactly as possible) and brain activation was measured not while participants executed the task but while they were informed that this was the task they were going to do next (as compared to another more boring task that was intermixed in the experiment). In the present study, rLPFC activation was measured *while* subjects were working on a task. Likely, these differences between the paradigms could have led to a difference in the location of LPFC activation between their and our task.

## Conclusion

Taken together, we can draw the following conclusions: (1) While administered, monetary rewards affected brain activation in response to feedback in the anterior striatum and midbrain. (2) After their withdrawal, verbal rewards affected brain activation in response to feedback in the anterior striatum and midbrain. (3) After their withdrawal, verbal rewards affected rLPFC activation while subjects worked on a picture puzzle task. Since we found no strong differences in behavioral task engagement, it is difficult to infer that these activation differences are indeed linked to intrinsic motivation or even task engagement. Possibly, intrinsic motivation in our task was not strong enough to be crowded-out by monetary rewards. The slight increase in correctly solved picture puzzles and the increased activation in our regions of interest in period 3 in the verbal reward group let us speculate that (low) intrinsic motivation was increased by verbal reinforcement. However, these allusions are not very strong and future research is essential to clarify the role of verbal reinforcement in intrinsic motivation.

### Conflict of interest statement

The authors declare that the research was conducted in the absence of any commercial or financial relationships that could be construed as a potential conflict of interest.
