# Supplementary material for: The brain correlates of the effects of monetary and verbal rewards on intrinsic motivation
Source: Front Neurosci. 2014 Sep 18;8:303. doi: 10.3389/fnins.2014.00303 (PMC4166960; doi:10.3389/fnins.2014.00303)
Supplement: Supplementary file 1 [file DataSheet1.DOCX]

**SUpplementary Material**

*Table A.1a.* Sidak-corrected behavioral results (post-hoc analysis) of 3 x 3 ANOVA with number of correct solved items as dependent variable.

|  |  | |  |  |  |
| --- | --- | --- | --- | --- | --- |
| *Group* | *Period* | | *Mean difference* | *Standard error* | *p* |
| Monetary | 1 | 2 | -1.909 | .893 | .106 |
|  | 1 | 3 | -0.591 | .838 | .862 |
|  | 2 | 3 | 1.318 | .828 | .311 |
| Verbal | 1 | 2 | -1.609 | .874 | .197 |
|  | 1 | 3 | -2.087 | .819 | .040 |
|  | 2 | 3 | -.478 | .810 | .913 |
| Control | 1 | 2 | -2.000 | .961 | .120 |
|  | 1 | 3 | -.368 | .901 | .968 |
|  | 2 | 3 | 1.632 | .891 | .201 |
|  |  |  |  |  |  |
| *Period* | *Group* | | *Mean difference* | *Standard error* | *p* |
| 1 | Monetary | Verbal | 1.565 | 1.264 | .526 |
|  | Monetary | Control | 1.684 | 1.327 | .506 |
|  | Verbal | Control | .119 | 1.314 | 1.000 |
| 2 | Monetary | Verbal | 1.866 | 1.021 | .202 |
|  | Monetary | Control | 1.593 | 1.072 | .369 |
|  | Verbal | Control | -.272 | 1.061 | .992 |
| 3 | Monetary | Verbal | .069 | 1.127 | 1.000 |
|  | Monetary | Control | 1.907 | 1.184 | .301 |
|  | Verbal | Control | 1.838 | 1.172 | .323 |

*Table A.1b.* Sidak-corrected behavioral results (post-hoc analysis) of ANOVA with fun ratings as dependent variable.

|  | | |  | | |  |  | |
| --- | --- | --- | --- | --- | --- | --- | --- | --- |
| *Group* | | | *Mean Difference* | | | *Standard error* | *p* | |
| Monetary | Control | | .4689 | | | .46870 | .687 | |
| Verbal | Control | | -.3730 | | | .46395 | .809 | |
| Monetary | Verbal | | .8419 | | | .44630 | .180 | |
|  | |  | |  |  | | |  |

*Table A.2*. Reported are Talairach coordinates of activation peaks in baseline period 1 for success minus failure feedback.

| *Success > failure feedback*  *(whole brain, p_FWE_ < .05)* | x | y | z | Statistical value | # of voxels |
| --- | --- | --- | --- | --- | --- |
| Ventral striatum | 9 | 8 | -11 | 6.58 | 29 |
| Ventral striatum | -12 | 5 | -11 | 6.21 | 23 |
| Middle occipital gyrus | -24 | -97 | 4 | 6.42 | 41 |
| Inferior occipital gyrus | -30 | -91 | -8 | 5.75 | 41 |
| Inferior occipital gyrus | -36 | -85 | -14 | 4.94 | 41 |
| Medial orbitofrontal gyrus | 3 | 47 | -11 | 5.72 | 29 |
| Superior frontal gyrus | -21 | 35 | 52 | 5.53 | 18 |
| Inferior parietal lobule | -45 | -67 | 40 | 5.24 | 8 |
| Postcentral gyrus | 15 | -34 | 64 | 5.15 | 3 |
| Precentral gyurs | 27 | -25 | 58 | 4.84 | 1 |
| Posterior cingulate gyrus | 6 | -55 | 19 | 4.96 | 1 |
| Middle occipital gyrus | 24 | -100 | 4 | 4.94 | 3 |
| Precentral gyrus | 57 | -1 | 7 | 4.89 | 2 |
| Middle frontal gyrus | -30 | 35 | -17 | 4.87 | 2 |
| Cingulate gyrus | -3 | -37 | 37 | 4.84 | 1 |
| *Success > failure feedback*  *(small volume corrected)* | x | y | z | Statistical value | # of voxels |
| Anterior Striatum | -18 | 14 | -8 | 3.26 (p_FWE_ = .063) | 2 |
| Midbrain | -6 | 5 | -11 | 5.98 (p_FWE_ < .05) | 21 |

*Table A.3*. Success - failure feedback. Reported are Talairach coordinates of activation peaks in our regions of interest (ROIs); (whole brain analysis; uncorrected, p < .001).

| *Monetary reward > control in period 2* | x | y | z | Statistical value | # of voxels |
| --- | --- | --- | --- | --- | --- |
| Anterior Striatum | 24 | 14 | 1 | 3.92 | 39 |
| Midbrain | -6 | -19 | -8 | 3.30 | 11 |
| *Monetary reward > control in period 2-1* | x | y | z | Statistical value |  |
| Anterior Striatum | 18 | 20 | -5 | 3.81 | 9 |
| Midbrain | -9 | -22 | 4 | 4.09 | 15 |
| *Verbal reward > control in period 3* | x | y | z | Statistical value |  |
| Anterior Striatum | 21 | 29 | -5 | 4.15 | 25 |
| Midbrain | -6 | -25 | -11 | 3.53 | 29 |
| *Verbal reward > control in period 3-1* | x | y | z | Statistical value |  |
| Anterior Striatum | 21 | 11 | -2 | 3.66 | 16 |
| Midbrain | -9 | -16 | -5 | 4.05 | 15 |

*Table A.4*. Picture presentation. Reported are Talairach coordinates of activation peaks in our regions of interest (ROIs); (whole brain analysis; uncorrected, p < .001).

| *Monetary reward > control in period 2* | x | y | z | Statistical value | # of voxels |
| --- | --- | --- | --- | --- | --- |
| No activation differences above threshold |  |  |  |  |  |
| *Monetary reward > control in period 2-1* | x | y | z | Statistical value |  |
| No activation differences above threshold |  |  |  |  |  |
| *Control > monetary reward in period 2* | x | y | z | Statistical value |  |
| rLPFC | 42 | 44 | 37 | 3.61 | 13 |
| *Control > monetary reward in period 2-1* | x | y | z | Statistical value |  |
| No activation differences above threshold |  |  |  |  |  |
| *Control > monetary reward in period 3* | x | y | z | Statistical value |  |
| No activation differences above threshold |  |  |  |  |  |
| *Control > monetary reward in period 3-1* | x | y | z | Statistical value |  |
| No activation differences above threshold |  |  |  |  |  |
| *Monetary reward > control in period 3* | x | y | z | Statistical value |  |
| rLPFC | 36 | 44 | 7 | 3.59 | 19 |
| rLPFC | 15 | 44 | 46 | 3.48 | 10 |
| *Monetary reward > control in period 3-1* | x | y | z | Statistical value |  |
| rLPFC | 21 | 20 | 46 | 4.45 | 50 |
| Verbal reward > control in period 3 | x | y | z | Statistical value |  |
| rLPFC | 42 | 8 | 46 | 3.92 | 16 |
| Verbal reward > control in period 3-1 | x | y | z | Statistical value |  |
| rLPFC | 60 | 35 | 1 | 3.94 | 17 |
| rLPFC | 24 | 17 | 37 | 3.34 | 10 |

*Table A.5*. Success – failure feedback. Reported are Talairach coordinates of activation peaks (whole brain analysis; p_FWE_ < .05).

| *Monetary reward > control in period 2* | x | y | z | Statistical value | # of voxels |
| --- | --- | --- | --- | --- | --- |
| No activation differences above threshold |  |  |  |  |  |
| *Monetary reward > control in period 2-1* | x | y | z | Statistical value |  |
| No activation differences above threshold |  |  |  |  |  |
| *Verbal reward > control in period 3* | x | y | z | Statistical value |  |
| Cingulate gyurs | 18 | 26 | 22 | 4.85 | 1 |
| *Verbal reward > control in period 3-1* | x | y | z | Statistical value |  |
| No activation differences above threshold |  |  |  |  |  |

*Table A.6*. Picture presentation. Reported are Talairach coordinates of activation peaks (whole brain analysis; p_FWE_ < .05).

| *Monetary reward > control in period 2* | x | y | z | Statistical value | # of voxels |
| --- | --- | --- | --- | --- | --- |
| No activation differences above threshold |  |  |  |  |  |
| *Monetary reward > control in period 2-1* | x | y | z | Statistical value |  |
| No activation differences above threshold |  |  |  |  |  |
| *Control > monetary reward in period 2* | x | y | z | Statistical value |  |
| No activation differences above threshold |  |  |  |  |  |
| *Control > monetary reward in period 2-1* | x | y | z | Statistical value |  |
| No activation differences above threshold |  |  |  |  |  |
| *Control > monetary reward in period 3* | x | y | z | Statistical value |  |
| No activation differences above threshold |  |  |  |  |  |
| *Control > monetary reward in period 3-1* | x | y | z | Statistical value |  |
| No activation differences above threshold |  |  |  |  |  |
| *Monetary reward > control in period 3* | x | y | z | Statistical value |  |
| rLPFC |  |  |  |  |  |
| *Monetary reward > control in period 3-1* | x | y | z | Statistical value |  |
| Temporal gyrus | 45 | -28 | 10 | 5.21 | 2 |
| Inferior parietal lobule | -51 | -52 | 37 | 5.07 | 1 |
| *Verbal reward > control in period 3* | x | y | z | Statistical value |  |
| No activation differences above threshold |  |  |  |  |  |
| *Verbal reward > control in period 3-1* | x | y | z | Statistical value |  |
| *No activation differences above threshold* |  |  |  |  |  |

*Table A.7*. Correlation of self-reported fun (on a 7-point Likert scale) and neural activation in our regions of interest.

| Group | Period | Brain region | Spearman’s rho | p |
| --- | --- | --- | --- | --- |
| Monetary | 3 | Midbrain | -.245 | .272 |
|  |  | Right striatum | .314 | .155 |
|  |  | Left striatum | .320 | .146 |
|  | 3-1 | Midbrain | -.190 | .396 |
|  |  | Right striatum | .259 | .245 |
|  |  | Left striatum | .176 | .433 |
| Verbal | 3 | Midbrain | .044 | .841 |
|  |  | Right striatum | .294 | .173 |
|  |  | Left striatum | .496 | .016 |
|  | 3-1 | Midbrain | .272 | .209 |
|  |  | Right striatum | -.006 | .978 |
|  |  | Left striatum | .208 | .341 |
| Control | 3 | Midbrain | .163 | .504 |
|  |  | Right striatum | -.094 | .703 |
|  |  | Left striatum | -.085 | .730 |
|  | 3-1 | Midbrain | .054 | .827 |
|  |  | Right striatum | .037 | .880 |
|  |  | Left striatum | .075 | .760 |
